# Enhancing the effects of nusinersen with cybernic treatment using Hybrid Assistive Limb (HAL) in spinal muscular atrophy: a real-world case series and exploratory cohort analysis

**DOI:** 10.1186/s13023-025-03681-9

**Published:** 2025-04-23

**Authors:** Takashi Nakajima, Toshio Saito, Akihiro Hashiguchi, Taiki Nakabayashi, Kazuki Kodera, Kota Utsumi, Takeshi Kanayama, Haruka Urabe, Satoru Kinoshita

**Affiliations:** 1 Department of Neurology, NHO Niigata National Hospital, Kashiwazaki, Japan; 2 Division of Child Neurology, Department of Neurology, NHO Osaka Toneyama Medical Center, Toneyama, Japan; 3 Department of Neurology and Geriatrics, Kagoshima University Graduate School of Medical and Dental Sciences, Kagoshima, Japan; 4 Department of Paediatrics, NHO Niigata National Hospital, Kashiwazaki, Japan

**Keywords:** Combined therapy, Cybernics, HAL, Hybrid assistive limb, Neurorehabilitation, Nusinersen, Spinal muscular atrophy

## Abstract

**Background:**

Nusinersen therapy for spinal muscular atrophy (SMA) provides significant functional improvement when initiated pre-symptomatically or early in life. However, challenges remain in diverse populations with longer disease duration. In Japan, innovative cybernic therapy with the Hybrid Assistive Limb (HAL) is gaining traction in treating neuromuscular diseases. This observational study investigated whether combining HAL therapy with nusinersen and conventional physiotherapy yields functional improvements in SMA, irrespective of disease duration or age. Functional improvement indicators included the Hammersmith Functional Motor Scale-Expanded (HFMSE) and Revised Upper Limb Module (RULM) scores, and the 2-minute walk test (2MWT), measured 15 months post-nusinersen initiation. A cohort analysis of a selected case series was conducted.

**Results:**

Twelve patients with SMA type 2 or 3 who met the criteria of being able to walk with a hoist and began nusinersen treatment > 40 months post-disease onset were selected for longitudinal clinical assessment. Cohort 1 (*n* = 5, mean age 36.0 years) underwent HAL therapy, while Cohort 2 (*n* = 7, 24.6 years) did not. Baseline characteristics, except mean age, were similar across cohorts. In Cohort 1, the period from baseline (nusinersen initiation) to HAL therapy ranged from 0 to 8.8 months. HFMSE scores improved in both cohorts at 15 months; the least squares mean (LSM) change from baseline (95% confidence interval [CI]) was 4.7 points (2.2, 7.3) in Cohort 1 and 2.9 points (0.7, 5.1) in Cohort 2. Clinically meaningful improvement of 3.0 points in HFSME was exceeded by four of five patients in Cohort 1 and three of seven in Cohort 2. The LSM change from baseline in RULM was 2.2 points (95% CI 1.0, 3.3) in Cohort 1, exceeding the minimal clinically important difference of 0.5–1.0 points, but remained unchanged in Cohort 2 due to ceiling effects (− 0.2; −1.5, 1.2; *p* = 0.016). The LSM change from baseline in the 2MWT had improved in Cohort 1 (34.57 m; 95% CI 4.57, 64.57), but not in Cohort 2 (− 3.86; −37.75, 30.03).

**Conclusions:**

In patients with SMA type 2 and 3, clinically meaningful improvements in multiple indicators were observed when HAL was combined with nusinersen, even when treatment commenced many years after disease onset.

**Registration:**

jRCT1090220400 (https://jrct.niph.go.jp/en-latest-detail/jRCT1090220400).

**Supplementary Information:**

The online version contains supplementary material available at 10.1186/s13023-025-03681-9.

## Background

Spinal muscular atrophy (SMA) is a rare genetic neuromuscular disease characterised by progressive motor dysfunction due to a loss of motor neurons within the anterior horn of the spinal cord [1, 2]. For many years, research has been conducted to elucidate the pathogenesis of SMA and develop disease-modifying therapies (DMTs) to halt its progression and to explore physical therapies to stabilise and improve functional impairment [3]. Combining these approaches has been increasingly recognised as a potentially ideal treatment strategy that synergistically enhances the therapeutic efficacy of each approach. However, evidence in this regard remains insufficient [3], highlighting the importance of accumulating data, particularly from real-world settings.

Over the past decade, the causative genetic mutation of SMA, as well as factors that partially determine disease severity and prognosis, along with epidemiological disease characteristics, have been clarified. SMA usually results from the homozygous absence of the survival motor neuron 1 (*SMN1*) gene on chromosome 5q13 [4]. *SMN1* and a nearly identical gene (*SMN2*) produce the SMN protein. *SMN1* codes for a full-length, fully functioning SMN protein, but a cytosine-to-thymine substitution in exon 7 of the *SMN2* gene results in an alternative splicing pattern that affects the SMN protein produced by this gene. As a result, almost all (90–95%) of the SMN protein from the *SMN2* gene is truncated, unstable and only partially functional, with only 5–10% being the functional isoform [1, 2]. Therefore, *SMN2* can partially, but not wholly, compensate for the lack of *SMN1* in patients with SMA. Epidemiological observations demonstrated that *SMN2* copy numbers vary among patients, and SMA severity is inversely correlated, but does not correspond perfectly, with copy number [5, 6]. These findings suggest that the number of *SMN2* copies influences clinical severity but that other neurophysiological factors may also be involved. It is possible that treatments aimed at functional restoration, such as physiotherapy, may impact these alternative neurophysiological factors.

In an epidemiological survey in Japan, the estimated number of patients with SMA in 2017 was 1,478, with a prevalence rate of 1.17 per 100,000 population and an incidence rate of 0.61 per 10,000 births [7]. Despite the relatively high incidence rate, the prevalence had remained low due to the poor prognosis associated with the disease, leading to its classification as a rare disease. Several types of SMA with different levels of clinical severity can occur, ranging from SMA type 0, which results in neonatal death, to SMA type 4, which usually develops with mild symptoms in adults and is associated with an average life expectancy [1, 2, 8]. Between these extremities are types 1, 2 and 3. SMA type 1 develops immediately after birth and is associated with muscle weakness (including being unable to sit without support) and difficulty in eating and breathing, requiring artificial nutritional and ventilatory support. SMA type 2 typically develops between 7 and 18 months after birth and is associated with muscle weakness; feeding difficulties are common in patients with SMA type 2 [9]. In addition, respiratory function is reduced [1, 2] and up to 39% of these patients may require respiratory support [10]. Patients with SMA type 2 are able to sit up but cannot stand or walk independently. SMA type 3 develops from 18 months to 30 years of age [1, 2]. Patients with SMA type 3 are initially able to stand and walk unaided, but progressive muscle weakness, including gait disturbance, leads to a gradual loss of ambulatory ability [1, 2].

In the last decade, DMTs for SMA have emerged, such as the antisense oligonucleotide drug nusinersen, which was developed to selectively modify the splicing of the *SMN2* pre-messenger ribonucleic acid (mRNA) to produce functional SMN protein [11]. This was followed several years later by risdiplam, a small molecule RNA splicing modifier that operates through a different mechanism of action [12]. In clinical practice, these DMTs are combined with conventional supportive therapies, such as nutritional therapy and assisted ventilation for patients with SMA type 1, as well as physiotherapy aimed at maintaining joint mobility and utilising residual motor function for all SMA types [1, 2].

In the ENDEAR [13] and CHERISH [14] phase 3 randomised clinical trials (RCTs), nusinersen significantly improved the achievement of motor milestones, event-free survival, and motor function in infants and children with SMA type 1 and 2 compared with control sham treatment [15, 16]. Based on these findings, nusinersen was approved in the United States (US), Europe and Japan in 2017. Significant clinical improvement can be achieved when nusinersen is administered early after the onset of the disease [13, 14] or in the pre-symptomatic stage [17]. In the CHERISH trial, nusinersen administration was associated with a least-squares mean difference in change compared with the control group of 4.9 points on the Hammersmith Functional Motor Scale-Expanded (HFMSE) [14], which far exceeded the minimal clinically important difference (MCID) of 3.0 points [14]. In particular, it was indicated in CHERISH that improvements in gross motor function were greater in younger children or those who received treatment earlier in their disease course [14].

In several real-world observational studies in patients with SMA types 2 and 3, nusinersen improved motor function in children and adults [18–22]. However, in two of these studies, improvements beyond a 3.0-point gain in HFMSE scores were limited to up to 50% of patients [19, 21], and in another, the mean change was < 3.0 points [22]. Similarly, in a meta-analysis of a diverse patient population that included adults and individuals with later-onset SMA (types 2 and 3), the improvement in HFMSE score did not reach 3.0 points [23]; subsequently, different cutoff points for clinical significance have been proposed from a large cohort study of untreated patients [24].

In order to improve outcomes, early use of DMTs, including pre-symptomatic treatment, and use in various populations and different SMA types at different stages after disease onset has been proposed. In addition, methods to improve functional outcomes with nusinersen treatment have been investigated. Indeed, a recent study in patients with SMA demonstrated that improvements in motor function are greater in those who received physiotherapy during nusinersen administration than in patients who received nusinersen alone [25]. Until recently, no successful RCTs assessing specific physiotherapy treatments for SMA have been reported, and combined strength and aerobic exercise training has not been actively practised due to the potential risk of harm from overuse [26]. However, in 2021, Nakajima and colleagues published the results of an RCT (NCY-3001) evaluating the efficacy and safety of a novel wearable cyborg Hybrid Assistive Limb (HAL), known as cybernic treatment, in patients with eight rare neuromuscular diseases [27, 28]. The trial enrolled 24 individuals, including five with SMA, and assessed walking endurance using the 2-minute walk test (2MWT) as the primary endpoint. This crossover study compared walking exercise therapy using HAL plus a hoist versus a hoist alone over nine sessions within a 13-week period. At the end of the first treatment phase, the 2MWT endurance showed a significantly greater improvement with HAL plus hoist compared with hoist alone (15.577%, *p* = 0.044), with respective increases of + 24.874% and + 9.297%. A crossover analysis further confirmed a significant treatment effect, with a mean 2MWT change of 10.066% (*p* = 0.037) compared with hoist alone. These findings contributed to the approval of cybernic treatment for neuromuscular diseases in Japan [27, 28] and the US [29].

HAL was developed as a robotic exoskeleton device that supports movement based on the patient’s voluntary motor intentions and allows motor learning while preventing overuse in patients with neuromuscular diseases [27, 28]. Cybernic treatment with HAL has effectively reduced serum creatine kinase [29–31], an indicator of muscle damage, and is well recognised as a functional therapeutic assistive device for incurable neuromuscular diseases. In contrast to a pharmacological approach to treatment that targets the cause of the disease (such as nusinersen), HAL promotes the optimisation of neuronal grouping and synaptic connections through neurorehabilitation, and more directly improves motor learning [27, 28]. Thus, motor function is improved via a mechanism independent of *SMN2* copy number and SMN production. Combining HAL with nusinersen is expected to have synergistic effects and has the potential to link the molecular pathological changes and neurophysiological changes induced by the drug-based treatment to more holistic and tangible functional changes. Walking exercise therapy utilising HAL is covered by general health insurance in Japan. However, its use is not universal across all medical institutions, even in Japan, due to the need for specialised rehabilitation facilities and staff training.

Whether therapy involves DMT or cybernic treatment using HAL, earlier initiation is likely to be more effective than later initiation. However, we undertook an observational study to examine the hypothesis that combining DMT (i.e., nusinersen) with cybernic treatment using HAL, along with conventional physiotherapy techniques, could improve motor function in SMA, even in patients who started nusinersen long after disease onset. We collected real-world data from a diverse population, regardless of SMA type, disease duration or patient age, to evaluate the impact of adding HAL therapy to conventional treatment (nusinersen plus individualised physiotherapy). In addition to data collection, we assessed individual patients within each cohort using key functional measures, including the HFMSE, revised version of the Upper Limb Module (RULM) and 2MWT, while also attempting cohort comparisons. Through this approach, we aimed to obtain pilot data on the effectiveness and safety of combining DMT with cybernic treatment using HAL, specifically in patients with SMA who were receiving nusinersen.

## Methods

### Design

This was a non-blinded, exploratory observational study consisting of a small case series conducted at three facilities in Japan: NHO Niigata National Hospital, NHO Osaka Toneyama Medical Centre and Kagoshima University Hospital. The study was performed in accordance with the Declaration of Helsinki. The study protocol complied with all applicable Strengthening the Reporting of Observational Studies in Epidemiology (STROBE) guidelines (see Supplementary material for a completed STROBE checklist) and Japanese local regulations, and was approved by the ethics committee of each facility. All adult patients provided written informed consent. Patients under the age of 15 years at enrolment were required to give the appropriate assent to participate, and their guardians provided written informed consent.

### Patients

Patients were eligible to participate if they were at least 100 cm tall, had a genetically confirmed diagnosis of SMA type 2, 3 or 4 at least 40 months before the start of treatment, were receiving conventional individualised physiotherapy and were either receiving or were candidates to receive nusinersen (Spinraza^®^; Biogen). To assess the effect of nusinersen on ambulation, the study included only patients who could stand and walk with a hoist or assistance but could not walk independently without an assistive device. Patients with SMA type 2 were included because a small number of these patients can stand/walk using a hoist support [7, 32]. There were no upper or lower age limits. Patients were also required to have the appropriate body dimensions (weight, height, leg length, waist width) to fit the HAL device. Patients were excluded if they had scoliosis, foot joint contractures or other structural deformities that may have impeded the use of HAL, or medical complications such as dyspnoea on exertion, heart failure, arrhythmia or myocardial infarction that may have made assessments difficult. As there are currently no data on the safety of nusinersen in pregnant or lactating women [33], these individuals were not eligible for inclusion, and female participants of childbearing potential were required to use adequate contraceptive measures for the duration of the investigation.

### Treatment

**Fig. 1:**
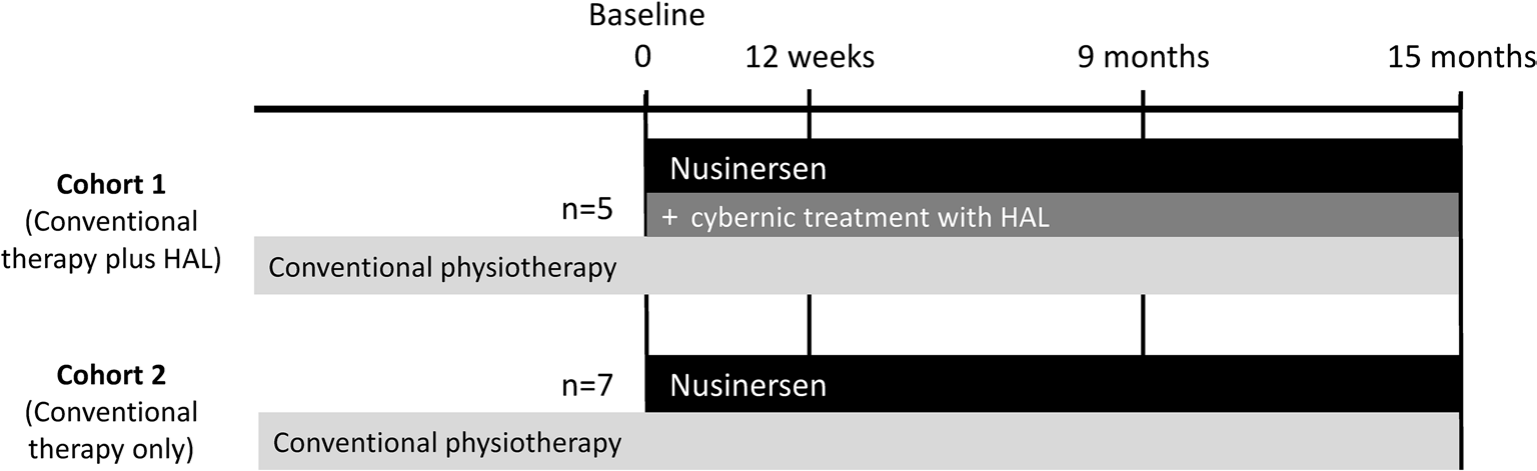
Investigation design. HAL, Hybrid Assistive Limb

Patients with SMA were administered nusinersen while receiving conventional individual-based physiotherapy. They were divided for observation into two cohorts; Cohort 1 (conventional therapy plus HAL) received nusinersen, conventional individual-based physiotherapy and cybernic treatment with HAL and Cohort 2 (conventional therapy only) received nusinersen and conventional individual-based physiotherapy only (without HAL; Fig. 1). The treatment assignment was not randomised; instead, selection to the cohorts was based on the availability of HAL for each patient at the centre where they were enrolled. Patients who wanted cybernic treatment were transferred to a facility where HAL was available. In both Cohorts 1 and 2, conventional physiotherapy was individualised to the patient’s conditions and needs, and administered according to the standard schedule used by the participating facility.

Cybernic treatment was administered according to standard practices [34]; walking exercise therapy with HAL (ML05 and FL06 2 S; Cyberdyne Inc., Tsukuba, Japan) commenced after fitting the device and adjusting the torque and signal sensitivity of the motor unit potentials from the skin. During cybernic treatment, a hoist (All in One^®^, Ropox A/S, Naestved, Denmark) was always used as a body weight support device during overground gait training for safety, to prevent falls. The duration of the walking exercise with HAL was 20–30 min/day, all in one session. The patient was generally encouraged to complete nine HAL sessions (per course) with a frequency of at least twice per week, following the guide for appropriate use of HAL [34]. In this observational study, we aimed to adhere to this recommendation as closely as possible. Multiple courses were recommended during the 15-month study period; however, as this was an observational study, no strict regulations were imposed beyond this recommendation. Nevertheless, the total number of HAL sessions, the total number of courses and the intervals between sessions were recorded for each patient.

All patients received nusinersen during the investigation period, which was administered according to the standard schedule used by the participating institutions. The approved dose of nusinersen in Japan for patients with later-onset SMA aged ≥ 2 years is 12 mg administered by intrathecal injection (5 mL volume) with three loading doses (Weeks 0, 4 and 12) followed by a maintenance dose every 6 months thereafter [35].

### Assessments

Assessments were made at baseline (before nusinersen administration), at Week 12 or Month 3 (end of the loading-dose period), at Month 9 (after the first maintenance dose of nusinersen) and at Month 15 (end of the investigation, after the second maintenance dose of nusinersen; Fig. 1). This schedule was aligned with the clinical trial timing [14] and post-marketing surveillance (PMS) [22] evaluations for nusinersen. The day on which the first dose of nusinersen was administered was designated as Day 0 for all timepoints. Because data were collected in a real-world setting, we allowed data collection within the following ranges for each timepoint: Baseline: as this was an observational study, baseline assessments were permitted from Day − 200 to Day 15, if necessary; Week 12: data collected between Day 16 and Day 157; Month 9: data collected between Day 158 and Day 374; and Month 15: data collected between Day 375 and Day 640. Where more than one assessment was observed within the relevant date range, the highest value was adopted. For patients already on nusinersen at enrolment, data on outcomes from the period before starting nusinersen were collected retrospectively from patients’ medical records, but all other data were collected prospectively after enrolment.

### Outcome measures

This observational analysis evaluated various motor function parameters, including changes in HFMSE total scores, RULM total scores, 2MWT, and other gait functions, as well as treatment regret, using the Decision Regret Scale (DRS).

The HFMSE is a scale that investigates the ability of a patient with SMA to perform various activities and has been validated in 2- to 45-year-old patients with SMA types 2 and 3 [36, 37]. The scale has 33 items, with each scored from 0 to 2. The maximum score is 66, which indicates the highest ability. The RULM is specifically designed to measure upper limb function in patients with SMA and is comprised of 19 items with a three-point grade system [38]. Its maximum total score of 37 points indicates the highest upper limb function. The 2MWT was used (with hoist support) to measure walking distance and indicate gait endurance [39]. A 10-metre walk test (10MWT; also with hoist support) was used to assess general walking functions [40, 41], including gait speed, cadence and step length. The test protocol included a 2-metre acceleration phase and a 2-metre deceleration phase, during which the patient was instructed to “go fast but not run”. The steady-state walking phase was maintained over the middle 6 m [40, 41], where gait speed, average step length and cadence were measured. The Japanese version of the DRS, a treatment-related patient-reported outcome, assessed patient satisfaction correlated with their treatment decisions, decisional conflict (feeling uncertain about the course of action taken) and overall rated quality of life [42, 43]. A score of 0 means no regret; a score of 100 means high regret [44].

### Statistical analyses

This observational study was designed first to analyse a case series and then to perform exploratory statistical analyses to the extent possible. It was not intended to test hypotheses regarding primary endpoints; instead, it attempted to compare two cohorts across all endpoints in an exploratory manner. However, when meaningful statistical testing was feasible, we aimed to determine the appropriate sample size for comparing the two cohorts. In the NCY-3001 study [27], the difference (standard deviation [SD]) in the rate of improvement between the HAL and the control groups after nine sessions (one course) for neuromuscular disease was 15.58% (19.1) in the 2MWT. When three or more courses were administered, the effect size was estimated to be at least 2.0, based on empirical findings indicating that the improvement rate was at least 2.5 times higher with continued treatment over three or more courses. Assuming a power of 90% and a significance level of 5%, the required sample size was calculated to be six per cohort, resulting in 12 patients overall. This sample size was deemed feasible for the study.

The data were analysed in the intent-to-treat population. Descriptive statistics were used, and the individual patient values and statistical summaries (mean, median) were provided for baseline characteristics and demographics, as well as the baseline values of the HFMSE, RULM and 2MWT. In Cohort 1, individual data and statistical summaries of cumulative use of HAL at 12 weeks (3 months), 9 months and 15 months were also provided. As an evaluation of the case series data, waterfall plots were generated for patients whose data were collected after 15 months. Missing values occurring within an allowed date range were imputed using data collected at the nearest subsequent assessment date to mitigate the risk of overestimating treatment effects in instances where efficacy was anticipated. When the baseline value was missing, the most recent value following the initiation of treatment was used as the baseline value. All outcome measures of Cohorts 1 and 2 were compared for change between baseline and time series (12 weeks [3 months], 9 months and 15 months) with mixed-effects models for repeated measures (MMRM). Changes from baseline in all motor function outcomes were summarised as the least squares mean (LSM) change, standard error of the mean (SEM) and 95% confidence intervals (CI). Results in Cohorts 1 and 2 were compared using MMRM at each time point, with the group, time point and group-time interaction as fixed effects for categorical variables, and baseline measurements as fixed effects for continuous variables as covariates. No additional imputation for missing values was made in the MMRM model. The significance level of the two-sided statistical tests was 5%. When comparing the two cohorts, the following patient attributes were considered confounding: type of SMA, age, period from onset of SMA to nusinersen administration, and the delay between nusinersen administration and cybernic treatment with HAL. Additionally, the number of walking exercise sessions with HAL was considered a confounding factor in assessing the effectiveness of cybernic treatment. These confounding factor data were described in the evaluation.

All statistical analyses were conducted with SAS version 9.4 or higher (SAS Institute; Tokyo, Japan).

## Results

### Patients

Twelve patients, comprising six children and six adults (five females and seven males) were sequentially enrolled between January 19, 2019, and December 26, 2019. Of these, five patients attended a medical facility where cybernic treatment with HAL was available (Cohort 1), and seven patients attended medical facilities where compatible HAL was unavailable (Cohort 2).

Patient demographics and baseline characteristics were generally similar between cohorts (Table 1). Both cohorts included children and adults (age range 9–65 years [Cohort 1] and 7–71 years [Cohort 2]). The mean (median) age was 36.0 (33) years in Cohort 1 and 24.6 (12) years in Cohort 2, with a tendency towards a slightly younger population in Cohort 2. The period from the SMA onset to nusinersen initiation ranged from 8.1 to 61.2 years in Cohort 1 and from 6 to 59.9 years in Cohort 2. The mean (median) duration between SMA onset and nusinersen initiation tended to be longer in Cohort 1 (30.3 [26.5] years) than in Cohort 2 (20.7 [10.1] years). Both cohorts had patients with SMA type 2 (Cohort 1, *n* = 2; Cohort 2, *n* = 2) and type 3 (Cohort 1, *n* = 3; Cohort 2, *n* = 5) disease. In both cohorts, the copy number of the *SMN2* gene was consistent with the SMA severity types.

**Table 1:** Patient demographics and baseline characteristics in cohorts 1 and 2

Patient identifier	Cohort 1 (plus HAL)						Cohort 2 (Conventional therapy only)							
	NH-01	NH-02	NH-03	NH-06	TH-01	Mean (median)	NH-04	NH-05	NH-07	TH-02	TH-03	KH-01	KH-02	Mean (median)
Sex	Female	Female	Female	Male	Male		Male	Male	Male	Male	Female	Female	Male	
Age, years	11	9	62	65	33	36.0 (33)	12	10	7	43	7	71	22	24.6 (12)
Age of SMA onset, years	1	1	1	19	6	5.6 (1)	1	1	1	6	1	11	6	3.7 (1)
Time from SMA onset to nusinersen administration, years	10	8.1	61.2	45.7	26.5	30.3 (26.5)	10.1	8.8	7.1	37	6	59.9	15.7	20.7 (10.1)
Type of SMA	2	2	3	3	3		3	3	2	3	2	3	3	
*SMN1* gene deletion	Y	Y	Y	Y	Y		Y	Y	Y	Y	Y	Y	Y	
*SMN2* copy numbers	2	3	4	4	3		3	3	3	3	3	4	4	
Height, cm	140	116	151	181	172.2	152.0 (151)	136.1	122.2	110	182	101	141.8	165.4	136.9 (136.1)
Body weight, kg	22	28.3	48.6	72.6	79	50.1 (48.6)	29.1	21.4	15	96.5	13.5	56.9	54.9	41.0 (29.1)
Previous HAL use	Y	N	Y	Y	N		N	N	N	N	N	N	N	
Time from BL to start of HAL, days	204	269	0	191	104		-	-	-	-	-	-	-	
BL motor function														
HFMSE total score, points	38	15	42	25	55	35.0 (38)	51	50	18	43	17	42	46	38.1 (43)
RULM total score, points	33	20	33	11	37	26.8 (33)	37	-	-	37	20	37	37	33.6 (37)
2MWT with hoist, m	30.66	8.17	26.16	37.64	102.30	35.97 (30.66)	50.75	55.50	-	47.10	-	93.00	-	61.59 (53.13)
Able to walk with hoist	Y	Y	Y	Y	Y		Y	Y	Y	Y	Y	Y	Y	
Usual mobility	EWC	EWC	Handrail	EWC	Handrail		WSOO	WSOO	EWC	Cane	EWC	Cane	Handrail	

2MWT, 2-minute walk test; BL, baseline; EWC, electric wheelchair; HAL, Hybrid Assistive Limb; HFMSE, Hammersmith Functional Motor Scale-Expanded; N, no; PT, physiotherapy; RULM, Revised Upper Limb Module; SMA spinal muscular atrophy; *SMN*, survival motor neuron (gene); WSOO, walking support outside only; Y, yes

There were also some differences between cohorts as to previous treatments and baseline motor function (Table 1). In Cohort 1, three patients had used the investigational or research model of HAL approximately nine times, about 4 years before this study, which may have elevated their baseline motor function. In contrast, no patients in Cohort 2 had prior experience with HAL. Baseline HFMSE total scores ranged from 15 to 55 points in Cohort 1, with a mean (median) of 35.0 (38.0) points, and were similar in Cohort 2, ranging from 17 to 51 points with a mean (median) of 38.1 (43.0) points. Baseline RULM scores ranged from 11 to 37 points in Cohort 1, with a mean (median) of 26.8 (33) points, while in Cohort 2, RULM scores ranged from 20 to 37 points, with a mean (median) of 33.6 (37) points, with four cases at the upper limit of 37 points. The baseline 2MWT distances were similar between cohorts, with a mean (median) of 35.97 (30.66) m in Cohort 1 and 61.59 (53.13) m in Cohort 2.

In Cohort 1, the time from baseline (nusinersen treatment) to receiving cybernic treatment with HAL ranged from 0 to 269 days (Table 1). At 15 months, patients in Cohort 1 received a mean (median) of 42.8 (36) sessions of HAL (Table 2). Since all patients received nusinersen, we analysed whether the combined therapy was adequately implemented for each patient based on the number of HAL sessions and their characteristics (Table 2). Although both NH-06 and TH-01 underwent three HAL courses, NH-06 had the fewest total HAL sessions (16 sessions), followed by TH-01. The mean session interval for NH-06 was 8.57 days within each course and 27.56 days throughout the entire treatment period, indicating a significantly lower session frequency. In contrast, the mean session interval for TH-01 was 3.03 days within each course and 15.0 days over the entire period, suggesting that the session intervals within each course were not notably prolonged. Based on these findings, NH-06 was the only patient for whom the combined therapy was not adequately implemented.

**Table 2:** Number of session and characteristics of HAL and 2MWT change in cohort 1

	Cohort 1 (plus HAL)						
Patient identifier	NH-01	NH-02	NH-03	NH-06	TH-01	Mean	(SD)
**Cumulative HAL use, number of sessions**							
3 months (12 weeks)	0	0	22	0	9	6.20	(9.65)
9 months	17	16	47	4	19	20.60	(15.88)
15 months	36	79	55	16	28	42.80	(24.71)
**Characteristics of HAL session**							
Total number of courses	4	5	6	3	3	4.20	(1.30)
Mean session interval entire treatment period (days)	9.00	4.20	8.45	27.56	15.00	12.84	(9.08)
Mean session interval within each course (days)	1.62	2.78	2.37	8.57	3.03	3.67	(2.79)
**2MWT change (m)**	20.56	24.36	46.69	-14.61	75.50	30.50	(33.39)

2MWT, 2-minute Walk Test; HAL, Hybrid Assistive Limb; SD, standard deviation

### Change in outcome measures

**Fig. 2:**
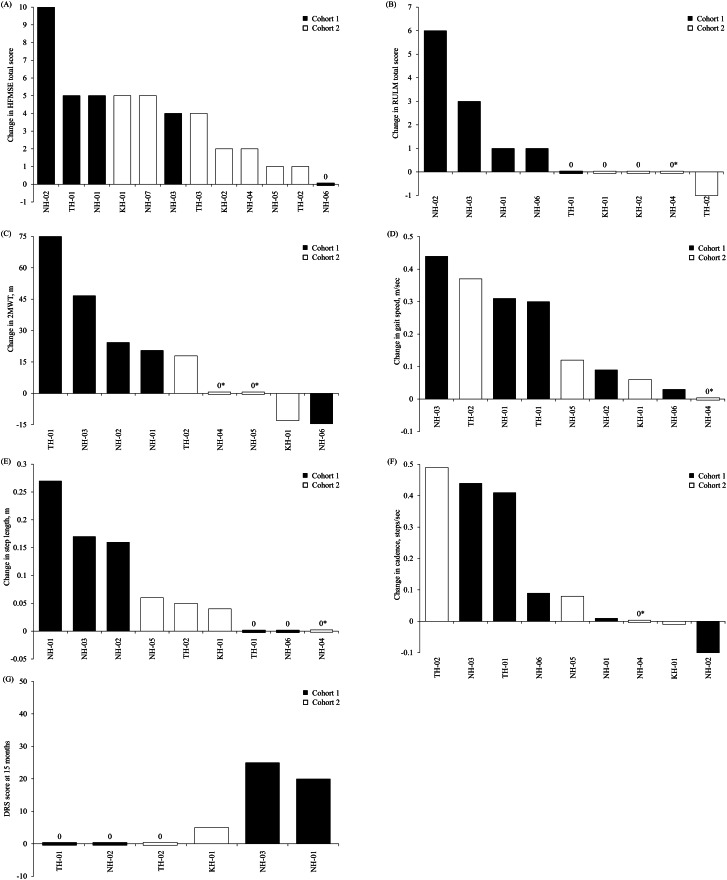
Waterfall plots of the mean change from baseline in each outcome measure at 15 months for (**A**) HFMSE total score, (**B**) RULM total score, (**C**) 2MWT, (**D**) gait speed, (**E**) step length and (**F**) cadence. (**G**) Waterfall plot for DRS score at 15 months. A “0*” denotes a solitary data point obtained overall, with the ensuing difference designated as zero. 2MWT, 2-minute walk test; DRS, Decision Regret Scale (Japanese version); HFMSE, Hammersmith Functional Motor Scale-Expanded; RULM, Revised Version of the Upper Limb Module

At 15 months, the HFMSE total score did not worsen in either cohort and 11 out of 12 patients showed improvement. In Cohort 1, four out of five patients had a clinically meaningful improvement (≥ 3 points), while in Cohort 2, three out of seven patients did (Fig. 2A). NH-06 underwent combined therapy with HAL but was the only patient who did not show improvement in the HFMSE score. The LSM change from baseline in HFMSE improved in both cohorts; by 4.7 points (95% CI 2.2, 7.3) in Cohort 1 and 2.9 points (95% CI 0.7, 5.1) in Cohort 2 (Table 3). The difference between cohorts in the change in HFMSE total score at 15 months was not significant (*p* = 0.254).

**Table 3:** Mixed-effects models for repeated measures^a^ analyses of the changes of all outcome measures at each time point from the baseline (nusinersen start time)

	Cohort 1 (Conventional therapy plus HAL)				Cohort 2 (Conventional therapy only)				Difference between cohorts		
	*n*	LSM	SEM	[95% CI for LSM]	*n*	LSM	SEM	[95% CI for LSM]	LSM	[95% CI for LSM]	*p*-value
HFMSE total score											
3 months (12 weeks)	5	1.7	1.06	[− 0.7, 4.1]	7	1.6	0.89	[− 0.4, 3.7]	0.1	[− 3.1, 3.3]	0.945
9 months	5	1.7	2.27	[− 3.3, 6.8]	7	1.1	1.92	[− 3.2, 5.3]	0.7	[− 6.0, 7.3]	0.826
15 months	5	4.7	1.13	[2.2, 7.3]	7	2.9	0.96	[0.7, 5.1]	1.8	[− 1.6, 5.2]	0.254
RULM total score											
3 months (12 weeks)	5	0.0	0.55	[− 1.2, 1.1]	5	0.0	0.55	[− 1.1, 1.2]	-0.1	[− 1.7, 1.6]	0.919
9 months	5	0.6	0.55	[− 0.6, 1.7]	5	−0.2	0.55	[− 1.3, 1.0]	0.7	[− 0.9, 2.4]	0.376
15 months	5	2.2	0.55	[1.0, 3.3]	4	−0.2	0.65	[− 1.5, 1.2]	2.3	[0.5, 4.2]	0.016
2MWT, m											
3 months (12 weeks)	5	28.19	12.19	[− 1.60, 57.98]	4	4.19	13.78	[− 29.48, 37.85]	24.01	[− 23.03, 71.04]	0.259
9 months	5	32.23	12.33	[2.27, 62.19]	4	3.51	13.93	[− 30.34, 37.36]	28.71	[− 18.54, 75.97]	0.189
15 months	5	34.57	12.28	[4.57, 64.57]	4	−3.86	13.88	[− 37.75, 30.03]	38.44	[− 8.88, 85.75]	0.094
10MWT											
Gait speed, m/sec											
3 months (12 weeks)	5	0.113	0.084	[− 0.090, 0.317]	4	0.071	0.094	[− 0.156, 0.298]	0.043	[− 0.262, 0.347]	0.747
9 months	5	0.207	0.093	[− 0.015, 0.430]	4	0.116	0.104	[− 0.133, 0.365]	0.092	[− 0.242, 0.425]	0.535
15 months	5	0.233	0.077	[0.048, 0.419]	4	0.138	0.086	[− 0.069, 0.346]	0.095	[− 0.183, 0.373]	0.44
Step length, m											
3 months (12 weeks)	5	0.029	0.030	[− 0.045, 0.104]	4	0.011	0.034	[− 0.072, 0.094]	0.019	[− 0.093, 0.130]	0.697
9 months	5	0.041	0.029	[− 0.029, 0.112]	4	0.021	0.032	[− 0.058, 0.099]	0.021	[− 0.085, 0.127]	0.647
15 months	5	0.121	0.039	[0.026, 0.217]	4	0.036	0.043	[− 0.071, 0.143]	0.086	[-0.058, 0.229]	0.194
Cadence, step/sec											
3 months (12 weeks)	5	0.136	0.105	[− 0.120, 0.392]	4	0.055	0.118	[− 0.235, 0.344]	0.081	[− 0.325, 0.487]	0.642
9 months	5	0.320	0.148	[− 0.038, 0.678]	4	0.100	0.167	[− 0.302, 0.502]	0.220	[− 0.328, 0.768]	0.37
15 months	5	0.190	0.114	[− 0.085, 0.466]	4	0.110	0.129	[− 0.201, 0.421]	0.080	[− 0.352, 0.512]	0.67

2MWT, 2-minute walk test; 10MWT, 10-rmetre walk test; CI, confidence interval; HAL, Hybrid Assistive Limb; HFMSE, Hammersmith Functional Motor Scale-Expanded; LSM, least squares mean; PT, physiotherapy; RULM, Revised Upper Limb Module; SEM, standard error of the mean

^a^In the analytical model, the group was defined as a fixed effect of the categorical variable. The analytical model included time point, group, and time point group interactions. Baseline measures were used as covariates as fixed effects for continuous variables. The Kenward-Roger method was used to estimate the degrees of freedom. The variance-covariance structure was assumed to be unstructured

RULM total scores showed a trend towards improvement from baseline at 15 months in Cohort 1, but not in Cohort 2. In Cohort 1, four out of five patients showed improvement in RULM total scores (Fig. 2B). Conversely, in Cohort 2, one out of five patients worsened. Although three patients in Cohort 2 at baseline had the maximum RULM score of 37, there was no deterioration, making further improvement undetectable due to the ceiling effect. The RULM total scores showed a trend towards improvement from baseline at 15 months in Cohort 1 (LSM change of 2.2; 95% CI 1.0, 3.3), but not in Cohort 2 (–0.2; 95% CI − 1.5, 1.2; Table 3). The difference between cohorts in the LSM change from baseline in RULM total score at 15 months was significant (2.3; 95% CI 0.5, 4.2; *p* = 0.016).

Of the nine patients with evaluable 2MWT scores at 15 months, five (four in Cohort 1 and one in Cohort 2) showed an increase, two (one in each cohort) demonstrated a decrease, and two patients in Cohort 2 each had a single measurement at 15 months, resulting in no change in value in the waterfall plot (Fig. 2C). NH-06 was the only patient in Cohort 1 who underwent combined therapy with HAL but did not show improvement in the 2MWT (Fig. 2C). Please see the Supplementary data, which includes video footage of one patient’s (NH-03) cybernic treatment with HAL (Additional file 1) and the change of 2MWT from baseline to 15 months (Additional file 2). At 15 months, 2MWT distance tended to increase from baseline in Cohort 1 (LSM change 34.57 m; 95% CI 4.57, 64.57) but not in Cohort 2 (− 3.86 m; 95% CI − 37.75, 30.03; Table 3). Despite observed improvements in 2MWT in Cohort 1 at 15 months, the between-group difference in 2MWT did not reach statistical significance (*p* = 0.094).

At 15 months, most patients had an improvement in gait speed, except for one patient in Cohort 2, who had no change in gait speed (Fig. 2D). Three patients in each cohort had improved from baseline in step length at 15 months (Fig. 2E), while three patients had no change in step length. At 15 months, four patients in Cohort 1 and two patients in Cohort 2 had improvements in cadence, one patient in Cohort 2 had no change in cadence and one patient in each cohort had a decrease in cadence (Fig. 2F). Comparison of LSM difference from baseline at 15 months showed an improvement in walking speed of 0.233 m/Sect. (95% CI 0.048, 0.419) and step length of 0.121 m/step (95% CI 0.026, 0.217) in Cohort 1 (Table 3). No significant differences were found between the cohorts.

Six patients had evaluable DRS scores at month 15: four in Cohort 1 and two in Cohort 2 (Fig. 2G). In both cohorts combined, the range of DRS scores was between 0 and 25, meaning that patients had little or no regret about their treatment.

## Discussion

This observational study demonstrated that the addition of cybernic treatment with HAL to nusinersen and conventional individual-based physiotherapy may enhance motor function and ambulation in patients with SMA type 2 or 3, even long after disease onset, when compared with those not receiving HAL. No significant adverse effects were observed in either cohort, and both cohorts demonstrated meaningful improvement in HFMSE, which is a key clinical outcome measure for gross motor function in SMA.

The LSM change in HFMSE from baseline to 15 months after the initiation of nusinersen was 4.7 points (95% CI 2.2, 7.3) in Cohort 1 and 2.9 points (95% CI 0.7, 5.1) in Cohort 2, with the lower limit of the 95% CI exceeding zero in both cohorts. Notably, LSM improvement in HFMSE surpassed the clinically meaningful threshold (≥ 3 points) in Cohort 1 and almost reached this criterion in Cohort 2, suggesting relevant improvement in both cohorts over 15 months. The results of this case series indicate a larger improvement compared with the PMS data of later-onset SMA in 151 patients in Japan who received nusinersen [22], where the mean (SD) change in the HFMSE was 1.9 (4.6) points from 16.7 (17.7) points at baseline to 18.6 (19.0) points at 15 months [22]. Our study found a marked improvement in HFMSE even with nusinersen monotherapy, particularly in Cohort 2, without using HAL. The mean (median) time from SMA onset to nusinersen administration in the PMS was 7572.8 (5171.0) days or 20.7 (14.17) years [22], which was not substantially different from our study of 30.3 (26.5) years in Cohort 1 and 20.7 (10.1) years in Cohort 2. However, unlike the PMS, our study required patients to be capable of walking with a hoist, which might explain the higher baseline HFMSE scores in our study (35.0 points in Cohort 1 and 38.1 points in Cohort 2) compared with the PMS baseline of 16.7 points [22]. Of note, comparisons between our study and the PMS should be made with caution, due to the small number of patients in our study, which limits the ability to compare baseline characteristics with those in the PMS population.

Our study observed a clinically meaningful improvement in the HFMSE (≥ 3 points [36, 37]) in four out of five cases in Cohort 1, and three out of seven cases in Cohort 2. One patient in Cohort 1 (NH-06) had the fewest HAL treatment sessions (16 sessions), with the longest average session interval within each course (8.57 days) and throughout the entire treatment period (27.56 days). This was the only patient in whom no improvement was observed in HFMSE, suggesting not only a diminished effect of the combined therapy but also a reduced effectiveness of nusinersen. Although Cohort 1 tended to show a greater improvement in HFMSE scores than Cohort 2, there was no significant difference between the cohorts. Nonetheless, if a new RCT is conducted based on the sample size determined using data from this study and if the combined therapy protocol is adhered to, it could provide more robust evidence.

In our study, the LSM change (95% CI) in RULM total score after 15 months was 2.2 (1.0, 3.3) in Cohort 1 and − 0.2 (− 1.5, 1.2) in Cohort 2. The lower limit of the 95% CI for Cohort 1 was above zero, and since the MCID for RULM is 0.5–1 [45], the LSM change of 2.2 in Cohort 1 exceeded the MCID, whereas that for Cohort 2 did not. The above data indicate that Cohort 1 demonstrated meaningful improvement in upper limb function.

While this result is encouraging, it is essential to note that MCID values in patients with SMA vary between ambulatory and non-ambulatory patients [45]. Studies have shown that the ceiling effect highly influences the MCID of RULM scores in ambulatory patients [45]. To appropriately evaluate the RULM, it is crucial to avoid ceiling effects with scores < 35 points and floor effects with scores > 10 points [46]. Although the mean baseline RULM score in Cohort 2 was 33.6 (i.e., within the > 10 and < 35 points range) in this study, four patients in Cohort 2 reached the maximum score of 37. However, caution is required when interpreting these results.

The LSM difference in the change in the RULM from baseline to 15 months between the cohorts was 2.33 (*p* = 0.016), indicating a statistically significant difference. Although caution should be taken in interpreting the statistical comparison between cohorts due to the considerations above, the significant change in RULM scores observed in Cohort 1 is clinically meaningful, according to the results of this study. One possible reason for the improvement in RULM is that gait training involves not only the lower limbs but also the trunk and upper limbs. Therefore, cybernic treatment with HAL may have contributed to the improvement in RULM scores. In addition to the physiotherapy approach required for using HAL, the increased frequency of visits to rehabilitation gyms may have led to a greater emphasis on upper limb rehabilitation, further contributing to the observed improvements. In this cohort study, the lack of strict definitions or frequency limitations on conventional physiotherapy approaches may have influenced the results. Moreover, no previous studies have specifically investigated the effects of cybernic treatment with the lower limb-type HAL on upper limb function in other disease populations, making it unclear whether this phenomenon is universally observed.

This study investigated the 2MWT with hoist support to indicate walking function, especially endurance. Generally, the 6-minute walk test (6MWT) has been recommended to evaluate walking function in patients with Duchenne muscular dystrophy (DMD) and SMA [47]. Historically, the MCID for the 6MWT in DMD has been considered to be 30 m [47]. It is thought to be similar in SMA, and studies of DMD and other neuromuscular diseases support the clinical meaningfulness of a 30-m improvement effect for the 6MWT [47, 48].

A notable feature of this study is that it included patients with SMA type 2 who could perform minimal walking movements with hoist assistance, making the 6MWT impossible to conduct in these patients, as it is conducted in DMD. In such cases, since the 2MWT is highly correlated with the 6MWT [39, 49, 50], it is possible to convert the 2MWT results to the 6MWT for evaluation in patients with low walking ability and susceptibility to fatigue.

Our analysis showed that in Cohort 1, the LSM change (95% CI) in the 2MWT distance from baseline to 15 months was 34.57 m (4.57, 64.57), with the lower limit of the 95% CI exceeding zero, indicating significant improvement. The MCID for the 2MWT in SMA has yet to be reported, but both the MCID and minimal important change for the 6MWT in SMA are considered to be 30 m. Using a conversion formula [49, 51], the equivalent change value for the 2MWT is approximately 10 m. In Cohort 1, four out of five patients showed an improvement of more than 10 m in the 2MWT. In contrast, NH-06, who had fewer HAL treatment sessions and longer session intervals than other patients in the cohort, was the only patient to show a deterioration of more than 10 m. In Cohort 2, one patient exceeded a 10-m improvement, and one showed a decrease of more than 10 m. The difference in LSM change between the two cohorts was 38.44 m (− 8.88, 85.75). Although the difference did not reach statistical significance in a two-tailed test (*p* = 0.094), data exceeding the estimated MCID of 10 m for the 2MWT is still considered clinically meaningful.

Improvement in the 2MWT is likely due to the combined effect of nusinersen and the addition of cybernic treatment with HAL. In previously reported PMS data [22], only 21 out of 275 patients could perform the 6MWT, with a mean (SE) improvement of 27.09 (9.81) m after nusinersen treatment, which did not reach the 30 m MCID for the 6MWT. In contrast, the mean change in the 6MWT reported in a European observational study was 30.86 mm (95% CI 18.34, 43.38) at 14 months, with 39 out of 89 patients (47.6%) exceeding 30 m [52]. The average improvement and proportion of patients showing improvement in Cohort 1 in our study far exceeded the abovementioned values for nusinersen monotherapy in the latter study.

Given that a DRS score of 25/100 is the cutoff value for clinical meaningfulness of low regret [42, 43], this analysis revealed that no patients in either cohort exceeded a DRS score of 25, indicating a low sense of regret in both cohorts. Patients could have participated in a different cohort if they had utilised a distant facility, as travel expenses were supported. However, each patient or their guardian ultimately made their selection considering the physical and time burdens of travel. Nonetheless, this suggests that patients in both cohorts were satisfied with the treatment effect and did not regret their participation. However, it should be noted that the DRS could only be assessed in a limited number of patients in this analysis (four in Cohort 1 and two in Cohort 2), and should, therefore, be interpreted with caution.

Overall, the results observed in Cohort 2 are consistent with previous observational studies showing the efficacy of nusinersen in patients with SMA type 2 or 3 [18–20, 53]. The results of Cohort 1 are consistent with data from the randomised NYC-3001 study, which showed that cybernic treatment with HAL was more effective than conventional physiotherapy at improving motor function in patients with neuromuscular disorders [27]. All patients in Cohort 1, except NH-06 who did not achieve successful combined therapy, showed improvements in both the 2MWT and HFMSE. This suggests that the quality of the combined therapy may be influenced by the total number of HAL sessions and the frequency of the session intervals.

There are several limitations to this study. When interpreting the differences between the two cohorts in this observational study, it is necessary to consider the impact of various biases or confounding factors. Unlike RCTs that control for confounding factors, the results of this observational study based on statistical analysis of a case series are dependent on the interpretation of confounding factors. Although we carefully conducted statistical comparisons between the two cohorts, the sample size of the study population remains relatively small and may, thus, have been influenced by various biases. Specifically, the ceiling effect in the RULM evaluation was a concern, and its potential impact was thoroughly discussed. Additionally, as this was a comparison of cohorts in an observational study and not an RCT, selection bias among the patients, particularly differences in the proportion of motivated patients between cohorts, was considered an inherent confounder that could not be analysed. Nevertheless, the purpose of this study was not to compare two treatments, as in an RCT, but to compare the addition of cybernic HAL therapy to conventional individual-based physiotherapy plus nusinersen treatment. Therefore, the differences in the method and amount of physiotherapy between the two cohorts were not an issue. Given the small number of patients with SMA in Japan [7, 32], the number of patients who met the inclusion criteria for recruitment within a reasonable time frame was limited, and the diversity of SMA phenotypes necessitated consideration of confounding factors during cohort comparisons [54]. However, in this study, the inclusion criteria that required the ability to walk with a hoist and a long duration from onset to nusinersen treatment, regardless of SMA type 2 or 3, resulted in a group with common walking impairment, mitigating this issue. In any case, the results and statistical data obtained from this investigation can be used to determine the sample size needed for future comparative trials to test hypotheses on primary endpoints. Upcoming studies should aim to standardise the definition of “conventional therapy”, as rehabilitation protocols can vary significantly.

This initial exploratory observational study is of significant importance because the results may provide sufficient data to inform clinical decision-making about the potential addition of cybernic treatment with HAL to treat patients with SMA, while simultaneously receiving nusinersen and standard individual-based physiotherapy. This approach may hold promise for other SMA therapies [55] that aim to increase SMN protein levels. However, while the study provided promising results, it does not establish the superiority of HAL therapy over other rehabilitation approaches. Additionally, confirmatory studies focusing on more homogeneous populations regarding SMA type, disease severity and age would be valuable.

## Conclusions

This case series of 12 patients with SMA receiving nusinersen and conventional individual-based physiotherapy suggested that adding cybernic treatment with HAL has the potential to further improve motor function in some patients. By dividing the cohorts based on whether cybernic HAL treatment was added, our analyses suggested that the addition of cybernic treatment may enhance motor function across diverse patients with SMA type 2 or 3; the cohort that received combined nusinersen with HAL showed clinically meaningful improvements in total HFMSE scores, total RULM scores and 2MWT distance at 15 months post-treatment, even many years after disease onset. Importantly, no significant adverse effects were observed, supporting the safety of this combined approach. Based on the findings from this study and our previous research, the therapeutic effects of nusinersen have the potential to be augmented when appropriate cybernic treatment is added to the treatment regime. This approach suggests that combining DMTs with cybernic treatment using HAL could improve functional outcomes in other neuromuscular disorders. However, further research is needed to identify the specific parameters where HAL demonstrates the greatest efficacy and to investigate differences in outcomes compared with various rehabilitation approaches. Future studies should also explore optimal patient selection criteria and long-term safety profiles to refine the integration of cybernic treatment into the standard management of patients with SMA.

## Electronic supplementary material

Below is the link to the electronic supplementary material.

**Supplementary Material 1:** Video clip 1 displays the initial cybernic treatment session using Hybrid Assistive Limb (HAL). NH-03, a 62-year-old female with SMA type 3, initially participated in body weight-supported treadmill training (BWSTT) with HAL for 20 min per session. Lateral (A) and frontal (B) views are shown simultaneously. As her walking pattern improved, she transitioned to body weight-supported overhead training (BWSOT) with HAL using a mobile hoist (video not shown). The filming and publication of this video were conducted with fully informed consent from NH-03

**Supplementary Material 2:** Video clip 2 demonstrates the first 30 s of the 2-minute walk test minute walk test (2MWT) for NH-03, showcasing improvements in walking pattern and speed from baseline (top) to 15 months later (bottom). The 2MWT distance also increased from 26.16 m at baseline (top) to 72.85 m 15 months later (bottom). NH-03 used a mobile hoist purely to prevent falls during the 2MWT evaluation. It is important to note that the hoist did not provide unloading of body weight, and the operator was not pulling; hence, the sling and harness were loose. The filming and publication of this video were conducted with fully informed consent from NH-03

**Supplementary Material 3:** STROBE Statement—checklist of items that should be included in reports of observational studies

## Data Availability

The datasets used and analysed during the current analysis are available from the corresponding author upon reasonable request.
